# Supplementary material for: A high-resolution, nanopore-based artificial intelligence assay for DNA replication stress in human cancer cells
Source: Nat Commun. 2025 Aug 19;16:7732. doi: 10.1038/s41467-025-63168-w (PMC12365011; doi:10.1038/s41467-025-63168-w)
Supplement: Supplementary file 1 — Supplementary Information [file 41467_2025_63168_MOESM1_ESM.pdf]

A high-resolution, nanopore-based artificial intelligence assay for DNA replication stress in human cancer cells

Mathew J.K. Jones[1,2,†], Subash Kumar Rai[1,], Pauline L. Pfuderer[7,9], Alexis Bonfim-Melo[1], Julia K. Pagan[3], Paul R. Clarke[1,4], Francis Isidore Garcia Totañes[6], Catherine J. Merrick[7], Sarah E. McClelland[5], Michael A. Boemo[7,8,*]

1. Frazer Institute, Faculty of Health, Medicine, and Behavioural Sciences, University of Queensland, Brisbane, QLD, 4072, Australia
2. School of Chemistry & Molecular Biosciences, University of Queensland, Brisbane, QLD, 4072, Australia
3. School of Biomedical Sciences, The University of Queensland, Saint Lucia, QLD 4072, Australia
4. Institute for Biomedicine and Glycomics, Griffith University, Southport, QLD 4222, Australia
5. Barts Cancer Institute, Queen Mary University of London, London, EC1M 6BQ, United Kingdom
6. Wellcome Sanger Institute, Cambridge, CB10 1SA, United Kingdom
7. Department of Pathology, University of Cambridge, Cambridge, CB2 1QP, United Kingdom
8. Department of Genetics, University of Cambridge, Cambridge, CB2 3EH, United Kingdom
9. Cancer Research UK Cambridge Centre, Li Ka Shing Centre, Robinson Way, Cambridge, CB2 0RE, United Kingdom

Correspondence: †mathew.jones@uq.edu.au, *mb915@cam.ac.uk

Figure S1

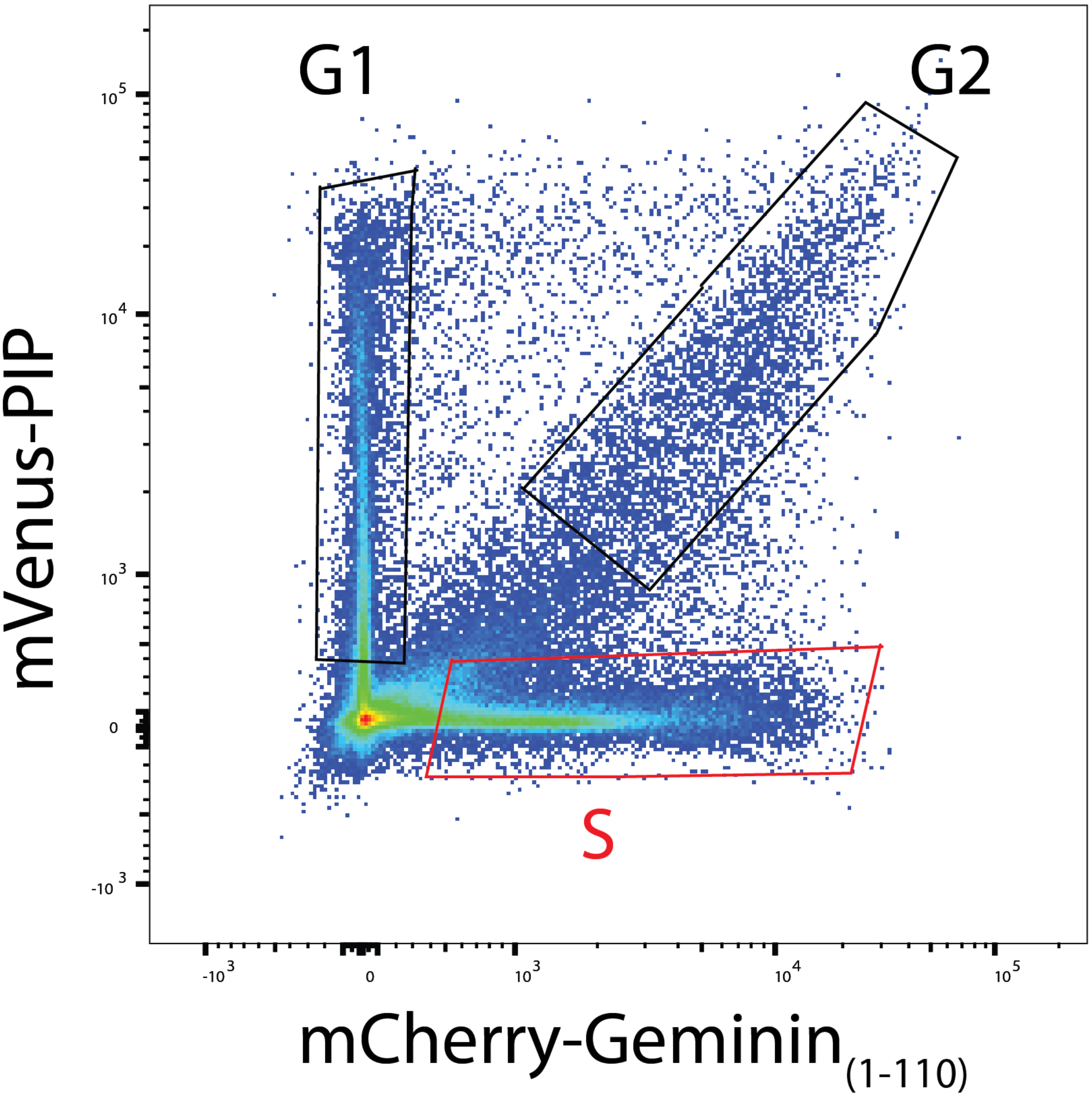

Figure S1: Sorting for S-phase cells using PIP-FUCCI. Representative plot of PIP-FUCCI sorting (A2058 cell lines treated with Olaparib for 24 hours) showing the S-phase gate (red box). The y-axis signal is mVenus-PIP and the x-axis mCherry-Geminin.

Figure S2

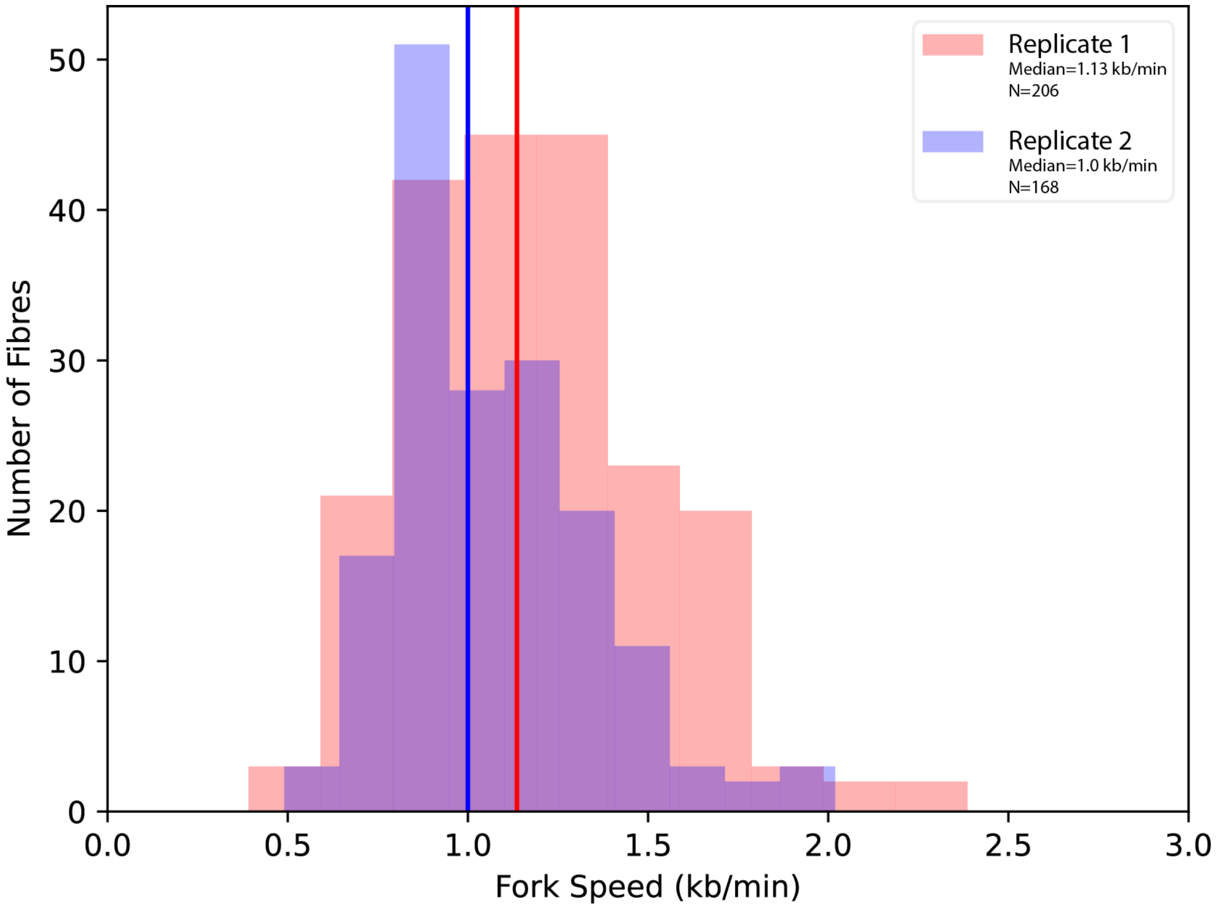

Figure S2: DNA fibre on A2058 cells. Two biological replicates of replication fork speed for A2058 cells as measured by DNA fibre. Vertical lines indicate the median for each replicate. Analogue track lengths (see Source Data) were each multiplied by a scaling factor of 2.58. Scaled IdU and CldU track lengths were then summed and divided by the total labelling time of 40 minutes. Source data are provided as a Source Data file.

Figure S3

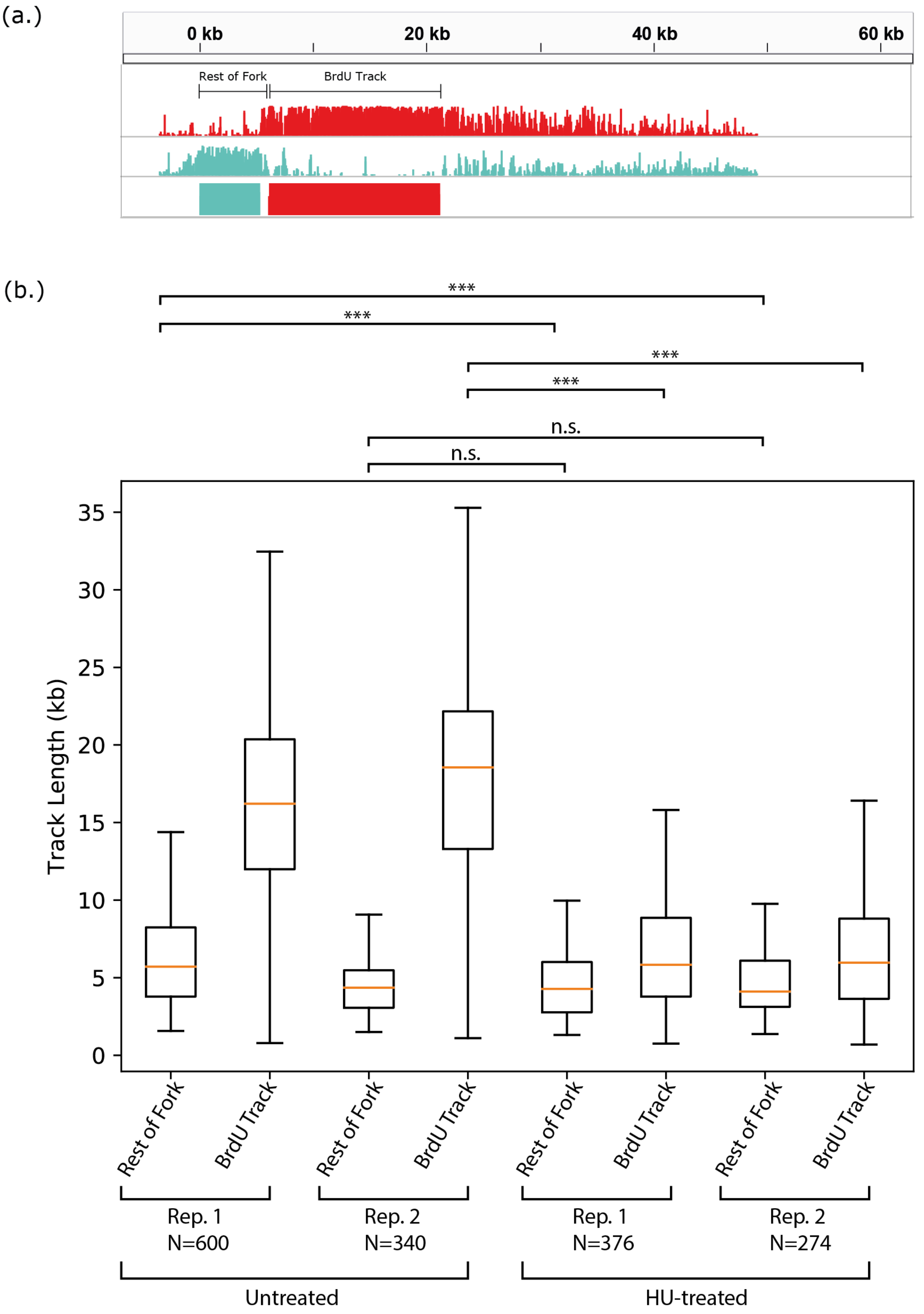

Figure S3: Comparison of analogue track lengths between untreated and HU-treated samples. (a.) Diagram indicating how track length was measured. (b.) Comparison of track lengths between untreated replicates and HU-treated replicates. Orange lines indicate the median. Track lengths were compared with a Mann-Whitney U test (n.s.=not significant; ***=p<0.001). Note that there is a small but significant (p<0.001) difference between the rest-of-fork track length in untreated Replicate 1 and that of the two HU-treated replicates. Source data are provided as a Source Data file.

Figure S4

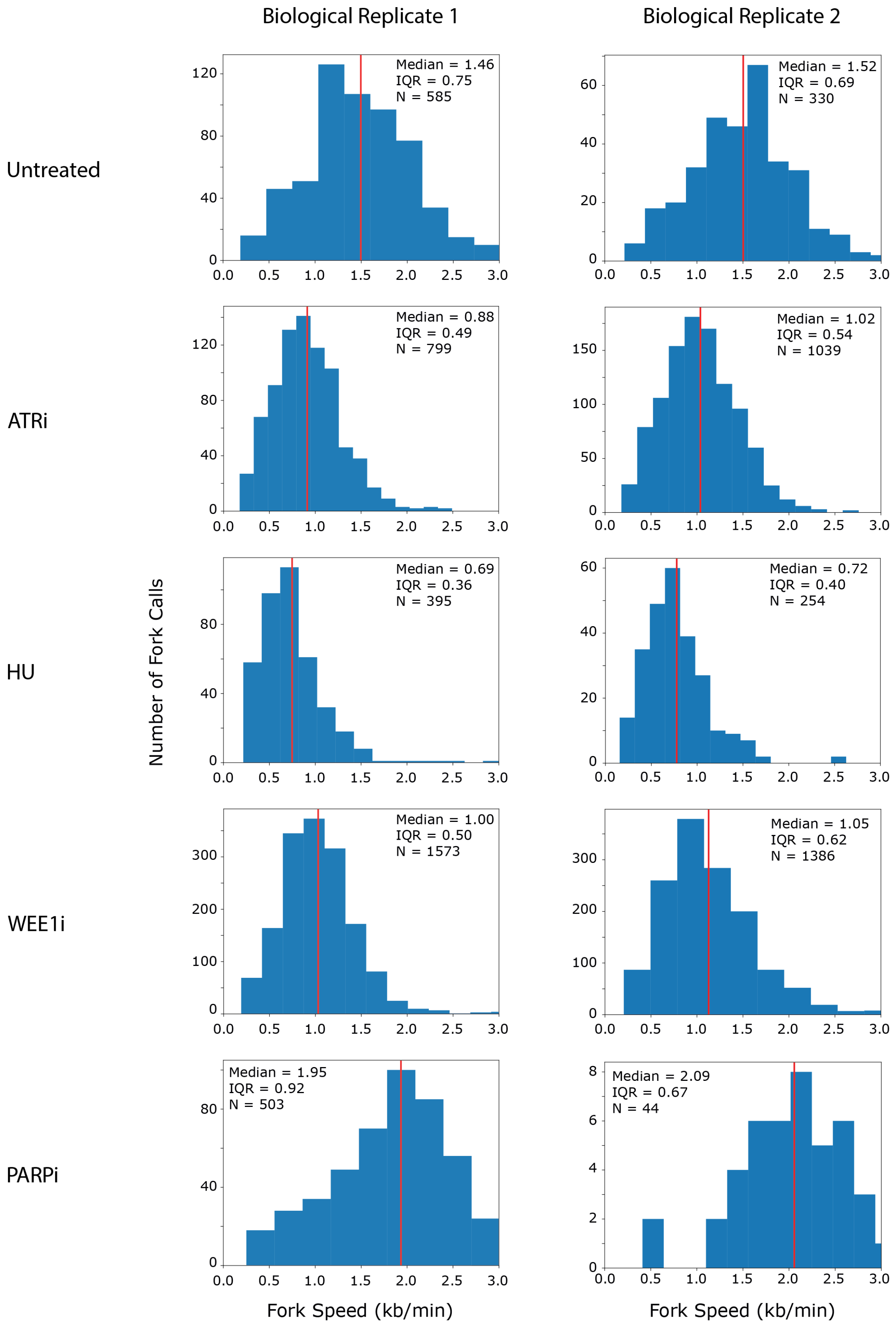

Biological Replicate 1
Biological Replicate 2
Untreated
ATRi
HU
WEE1i
PARPi
Number of Fork Calls
Fork Speed (kb/min)
Fork Speed (kb/min)
Median = 1.46
IQR = 0.75
N = 585
Median = 1.52
IQR = 0.69
N = 330
Median = 0.88
IQR = 0.49
N = 799
Median = 1.02
IQR = 0.54
N = 1039
Median = 0.69
IQR = 0.36
N = 395
Median = 0.72
IQR = 0.40
N = 254
Median = 1.00
IQR = 0.50
N = 1573
Median = 1.05
IQR = 0.62
N = 1386
Median = 1.95
IQR = 0.92
N = 503
Median = 2.09
IQR = 0.67
N = 44

Figure S4: Measured replication fork speed is consistent across biological replicates. The distribution of fork speeds are shown for two biological replicates (columns) of each treatment (rows). The left column is shown in Figure 2c of the main text. Vertical red line shows the median, and the value of the median, number of fork calls in the distribution (N), and interquartile range (IQR) are shown for each replicate. Source data are provided as a Source Data file.

Figure S5

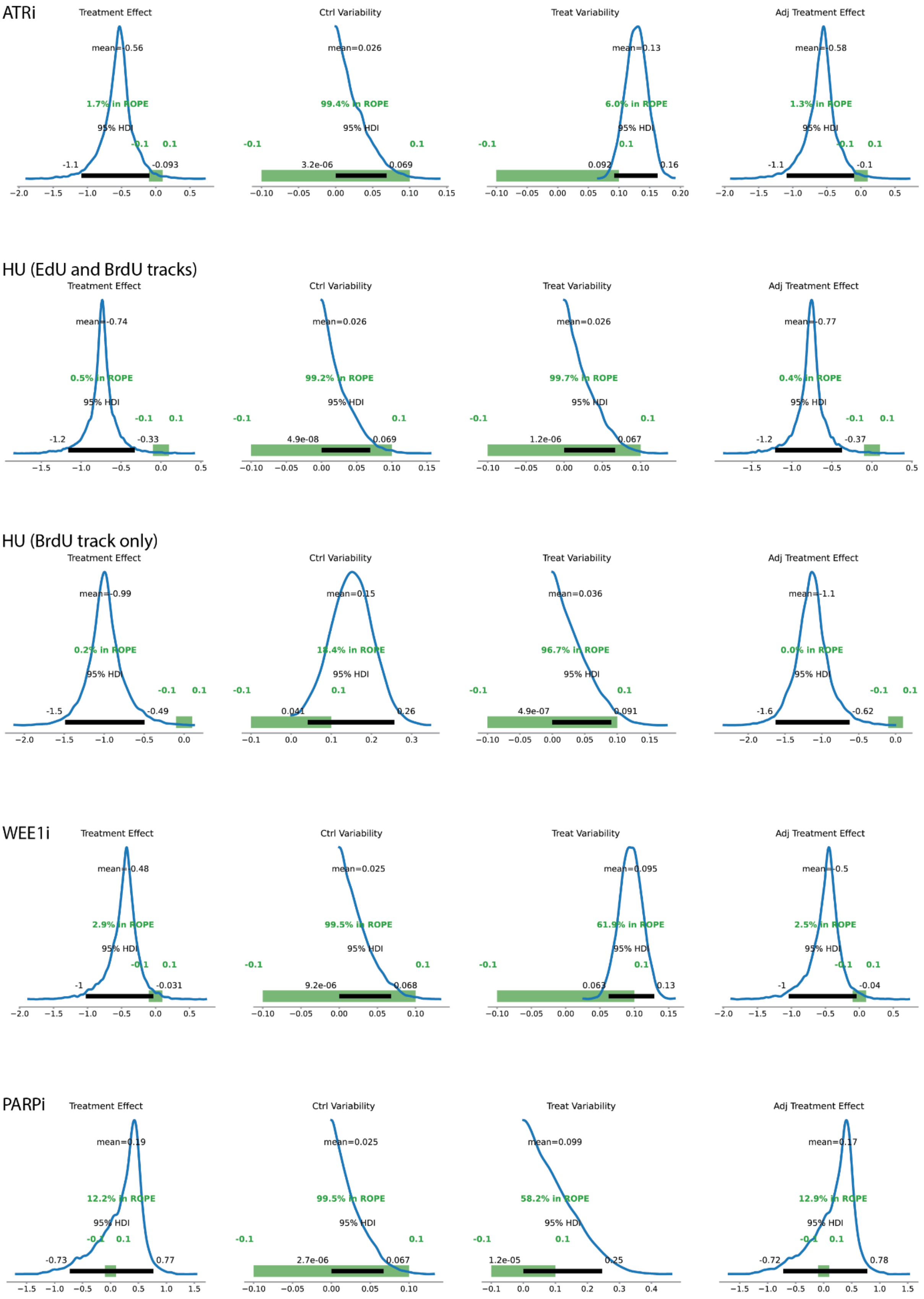
ATRi
Treatment Effect
mean=-0.56
1.7% in ROPE
95% HDI
-1.1
-0.093
Ctrl Variability
mean=0.026
99.4% in ROPE
3.2e-06
0.069
Treat Variability
mean=0.13
6.0% in ROPE
0.092
0.16
Adj Treatment Effect
mean=-0.58
1.3% in ROPE
-1.1
-0.1
HU (EdU and BrdU tracks)
Treatment Effect
mean=-0.74
0.5% in ROPE
-1.2
-0.33
Ctrl Variability
mean=0.026
99.2% in ROPE
4.9e-08
0.069
Treat Variability
mean=0.026
99.7% in ROPE
1.2e-06
0.067
Adj Treatment Effect
mean=-0.77
0.4% in ROPE
-1.2
-0.37
HU (BrdU track only)
Treatment Effect
mean=-0.99
0.2% in ROPE
-1.5
-0.49
Ctrl Variability
mean=0.15
18.4% in ROPE
0.041
0.26
Treat Variability
mean=0.036
96.7% in ROPE
4.9e-07
0.091
Adj Treatment Effect
mean=-1.1
0.0% in ROPE
-1.6
-0.62
WEE1i
Treatment Effect
mean=-0.48
2.9% in ROPE
-1
-0.031
Ctrl Variability
mean=0.025
99.5% in ROPE
9.2e-06
0.068
Treat Variability
mean=0.095
61.9% in ROPE
0.063
0.13
Adj Treatment Effect
mean=-0.5
2.5% in ROPE
-1
-0.04
PARPi
Treatment Effect
mean=0.19
12.2% in ROPE
-0.73
0.77
Ctrl Variability
mean=0.025
99.5% in ROPE
2.7e-06
0.067
Treat Variability
mean=0.099
58.2% in ROPE
1.2e-05
0.25
Adj Treatment Effect
mean=0.17
12.9% in ROPE
-0.72
0.78

Figure S5: Treatment effect on fork speed. The results of a hierarchical Bayesian model on the replication fork speed data in Figure 2c and Figure S4 indicating whether the change in replication fork speed due to treatment is higher than would be expected given the variation between replicates. Priors and sampling are as described in Methods ("Treatment effect"). From left to right, plots are treatment effect (Δ_1, the difference between group mean of treated and the group mean of untreated), control replicate variability (Δ_2, the absolute value of the difference between the untreated replicate means), treated replicate variability (the absolute value of the difference between the treated replicate means), and adjusted treatment effect (Δ_1 -Δ_2). For replication fork speed, we defined a Region of Practical Equivalence (ROPE) of ±0.1 kb/min. The ROPE (green) is shown together with the Highest Density Interval (HDI), the narrowest interval that contains 95% of the probability mass of the posterior. As shown in Figure 2a, HU was delivered together with the BrdU pulse. The row labelled "HU (EdU and BrdU tracks)" shows the results using the fork length as shown at the top of Figure 2b by taking the distance between the start of the EdU track and the end of the BrdU track for both HU-treated and untreated forks. The row labelled "HU (BrdU track only)" uses the same hierarchical Bayesian analysis but only uses the length of the BrdU track for both HU-treated and untreated forks.

Figure S6

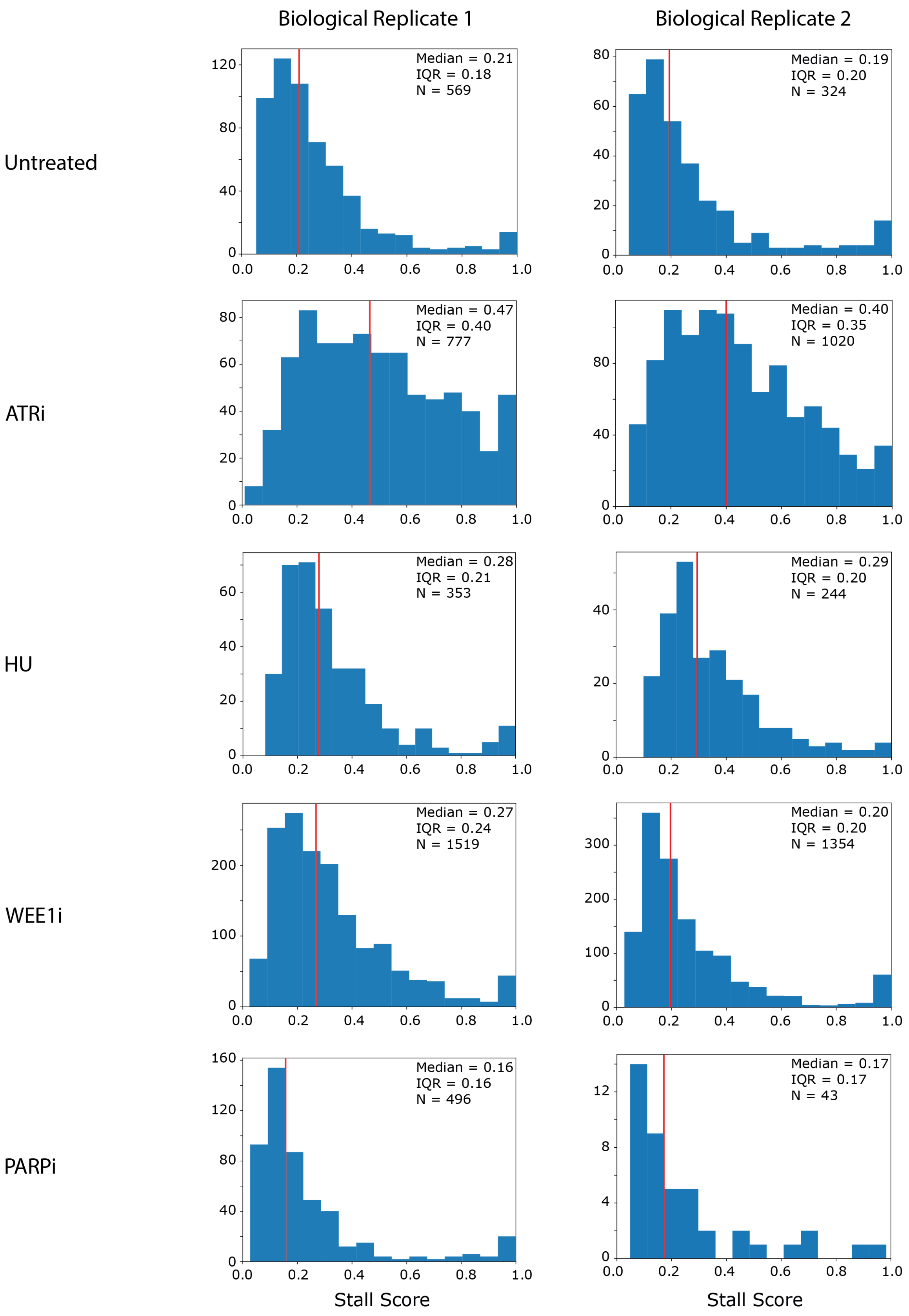

Biological Replicate 1
Biological Replicate 2
Untreated
ATRi
HU
WEE1i
PARPi
Median = 0.21
IQR = 0.18
N = 569
Median = 0.19
IQR = 0.20
N = 324
Median = 0.47
IQR = 0.40
N = 777
Median = 0.40
IQR = 0.35
N = 1020
Median = 0.28
IQR = 0.21
N = 353
Median = 0.29
IQR = 0.20
N = 244
Median = 0.27
IQR = 0.24
N = 1519
Median = 0.20
IQR = 0.20
N = 1354
Median = 0.16
IQR = 0.16
N = 496
Median = 0.17
IQR = 0.17
N = 43
Stall Score
Stall Score

Figure S6: Measured stall score is consistent across biological replicates. Similar to Figure S4, but showing the distribution of stall scores for two biological replicates (columns) of each treatment (rows). Source data are provided as a Source Data file.

Figure S7

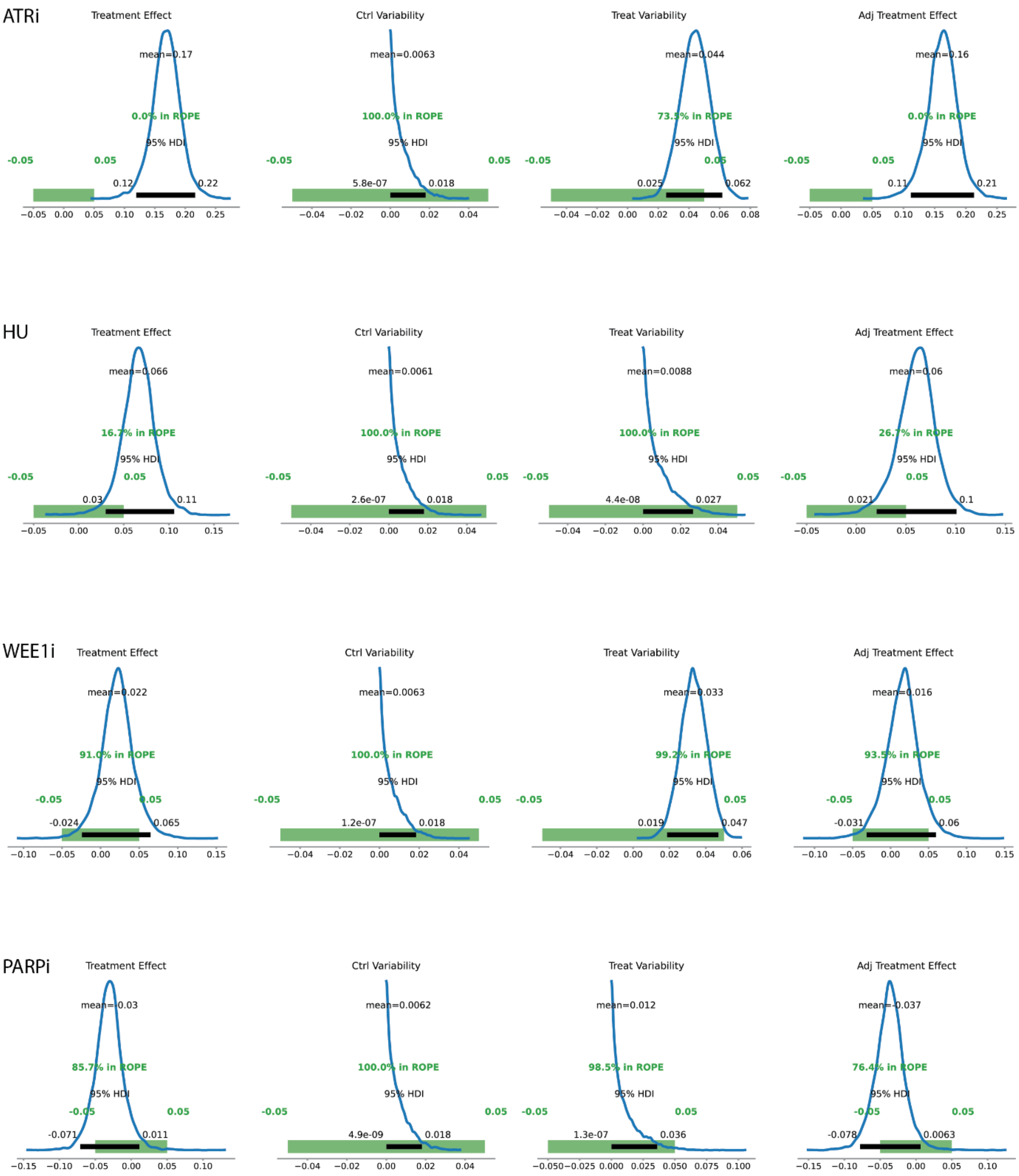

.

Figure S7: Treatment effect on stall score. Similar to Figure S5, but showing the treatment effect of the stall scores from Figure 3b and Figure S6. For stall score, the ROPE is defined as ±0.05.

Figure S8

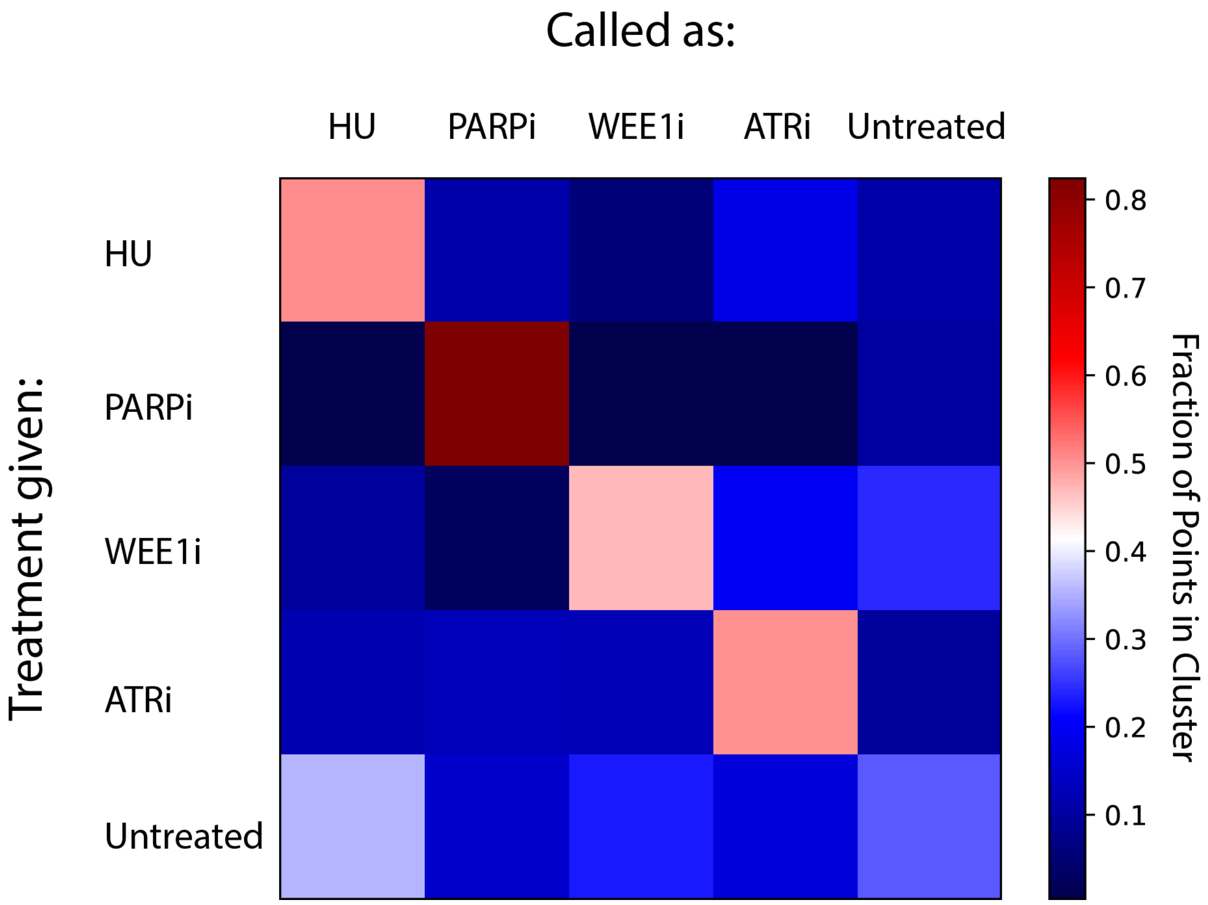

Figure S8: Confusion matrix of replication signatures. The points in Figures 3c were partitioned by k-means clustering (k=5). As shown by the centroids in Figure 3c, there is one cluster for each of the four treatments along with an additional cluster for the untreated case. Each row indicates a k-means cluster that corresponds to a treatment. Columns show the fraction of forks in that cluster that were given each treatment.

Figure S9

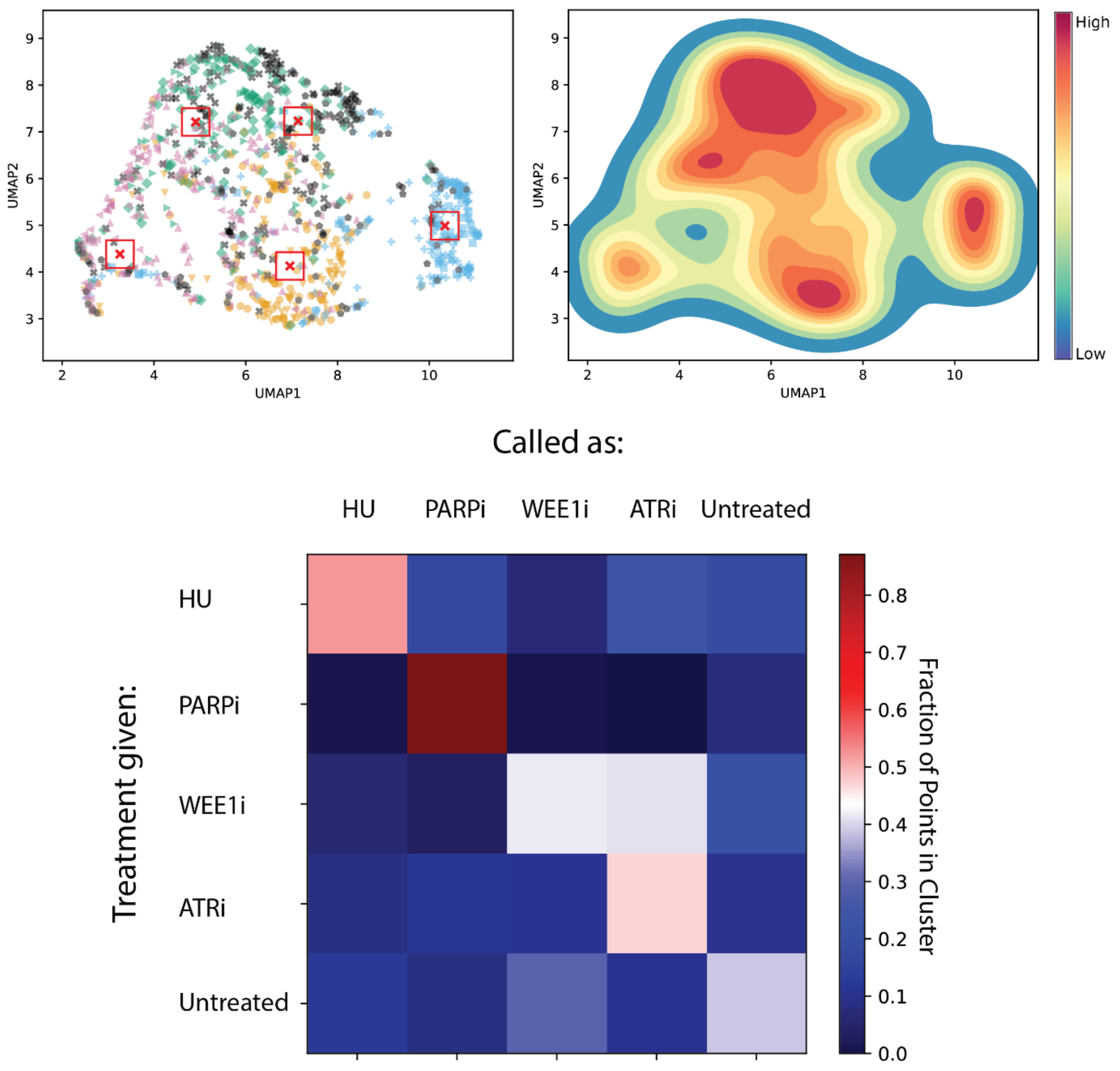

Figure S9: Replication signatures using the BrdU track alone. To account for the fact that HU was added together with the BrdU pulse as shown in Figure 2a, we repeated the analysis shown in Figure 3c and Figure S8 using the length of the BrdU track as the only metric of fork speed in the replication stress signature. Note that doing so turns the 8-dimensional points into 6-dimensional points. In particular, the features are: (i.) the length of the BrdU track (in bp), (ii.) fraction of thymidine positions called as BrdU in the EdU segment, (iii.) fraction of thymidine positions called as EdU in the EdU segment, (iv.) fraction of thymidine positions called as EdU in the BrdU segment, (v.) fraction of thymidine positions called as BrdU in the BrdU segment, (vi.) the stall score. Source data are provided as a Source Data file.

Figure S10

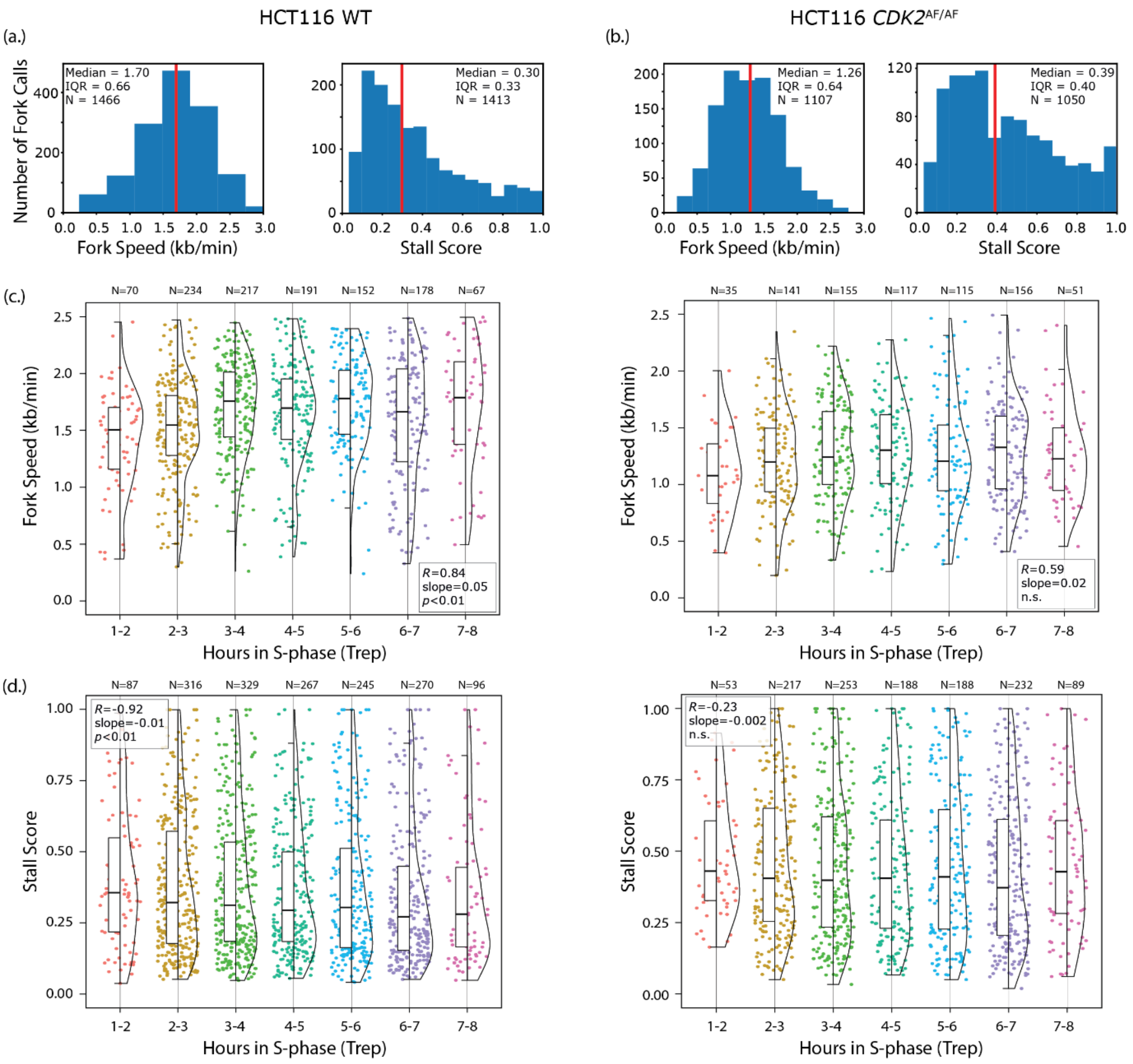

Figure S10: Changes in replication stress dynamics over S-phase are consistent across biological replicates. Biological replicate of the experiments shown in Figure 4 of the main text. (a.) Distribution of fork speeds and stall scores for HCT116 wild-type cells. (b.) Distribution of fork speeds and stall scores for the HCT116 *CDK2*$^{AF/AF}$ mutant. (c.) Distribution of fork speeds and (d.) stall scores of forks grouped by the median replication time (Trep) of their genomic position. The statistics were computed as detailed in the caption of Figure 4. Source data are provided as a Source Data file.

Figure S11

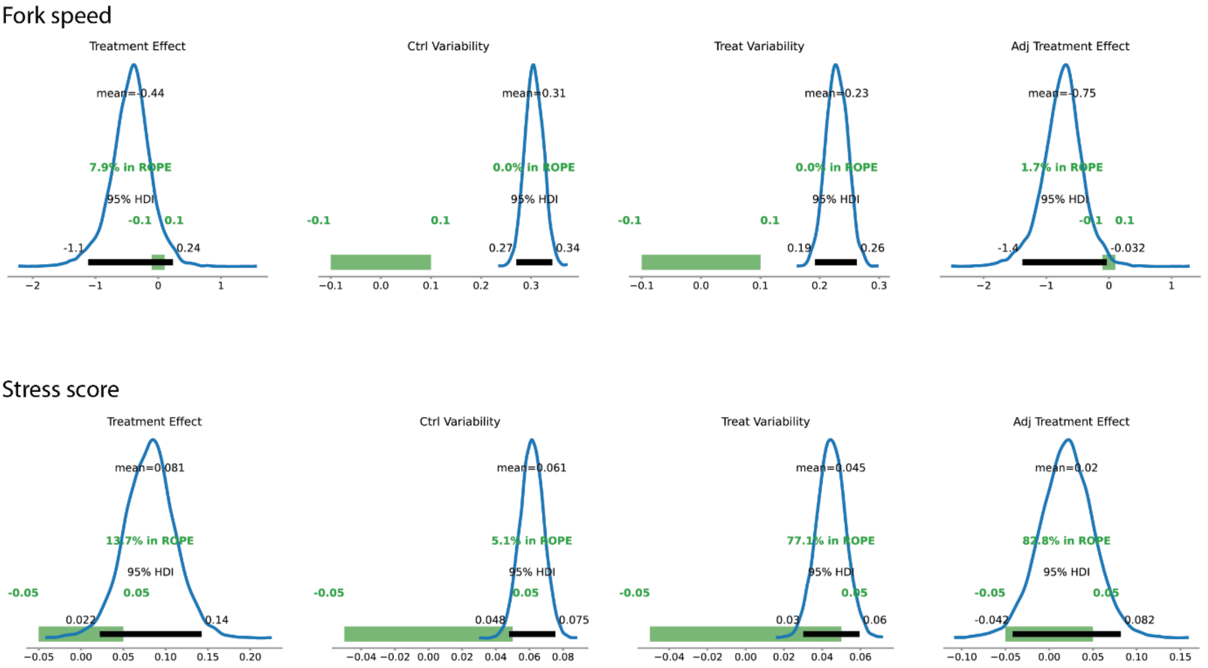

Figure S11: *CDK2*$^{AF/AF}$ effect on fork speed and stall score. Similar to Figures S5 and S7, but showing the effect of the *CDK2*$^{AF/AF}$ on replication fork speed and stall score using the replicates from Figure 4 and Figure S10.

Figure S12

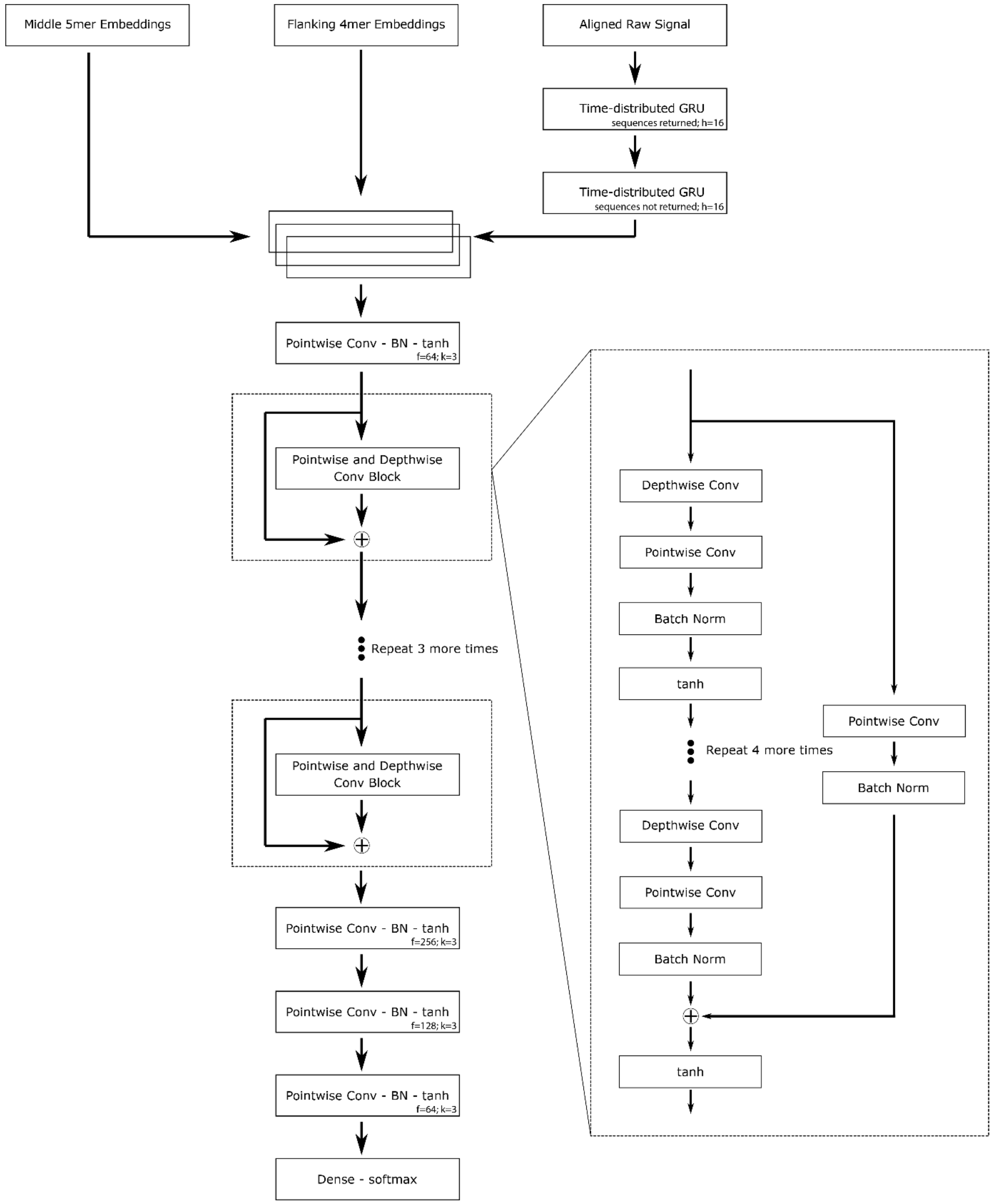

Figure S12: Neural network architecture for DNAscent v4.0.3. Neural network architecture for the model used to call BrdU and EdU on R10.4.1 chemistry in DNAscent v4.0.3. BN=batch normalisation; h=size of hidden state; f=number of filters; k=size of the 1D convolution kernel. Table S3 shows the values of the number of filters and kernel size for each residual block.

Figure S13

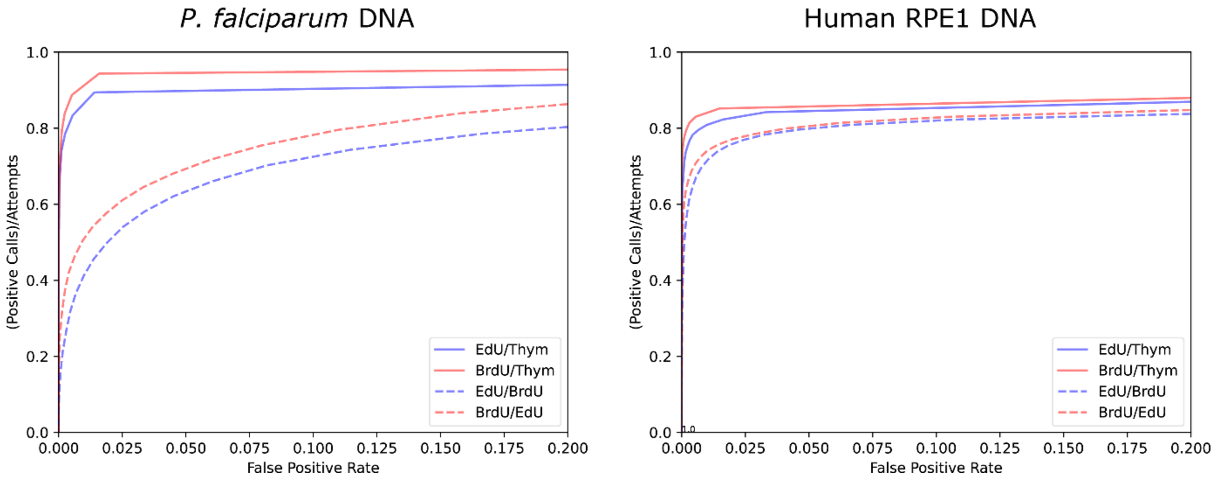

Figure S13: Receiver operator characteristic (ROC) curves for DNAscent v4.0.3. Curves comparing the fraction of positive calls (y-axis) to the false positive rate (x-axis) for different probability thresholds above which the neural network's call is considered positive (points along the curve). Curves show model performance on BrdU-labelled DNA with unlabelled DNA used to measure false positives (solid red curve), EdU-labelled DNA with unlabelled DNA used to measure false positives (solid blue curve), BrdU-labelled DNA with EdU-labelled DNA used to measure false positives (dashed red curve), and EdU-labelled DNA with BrdU-labelled DNA used to measure false positives (dashed blue curve). Human RPE1 curves were computed on 200 2-kilobase test segments for each of the EdU-labelled, BrdU-labelled, and unlabelled conditions. *Plasmodium falciparum* curves were computed on 200 2-kilobase test segments for each conditions. None of these read segments were used in model training.

Figure S14

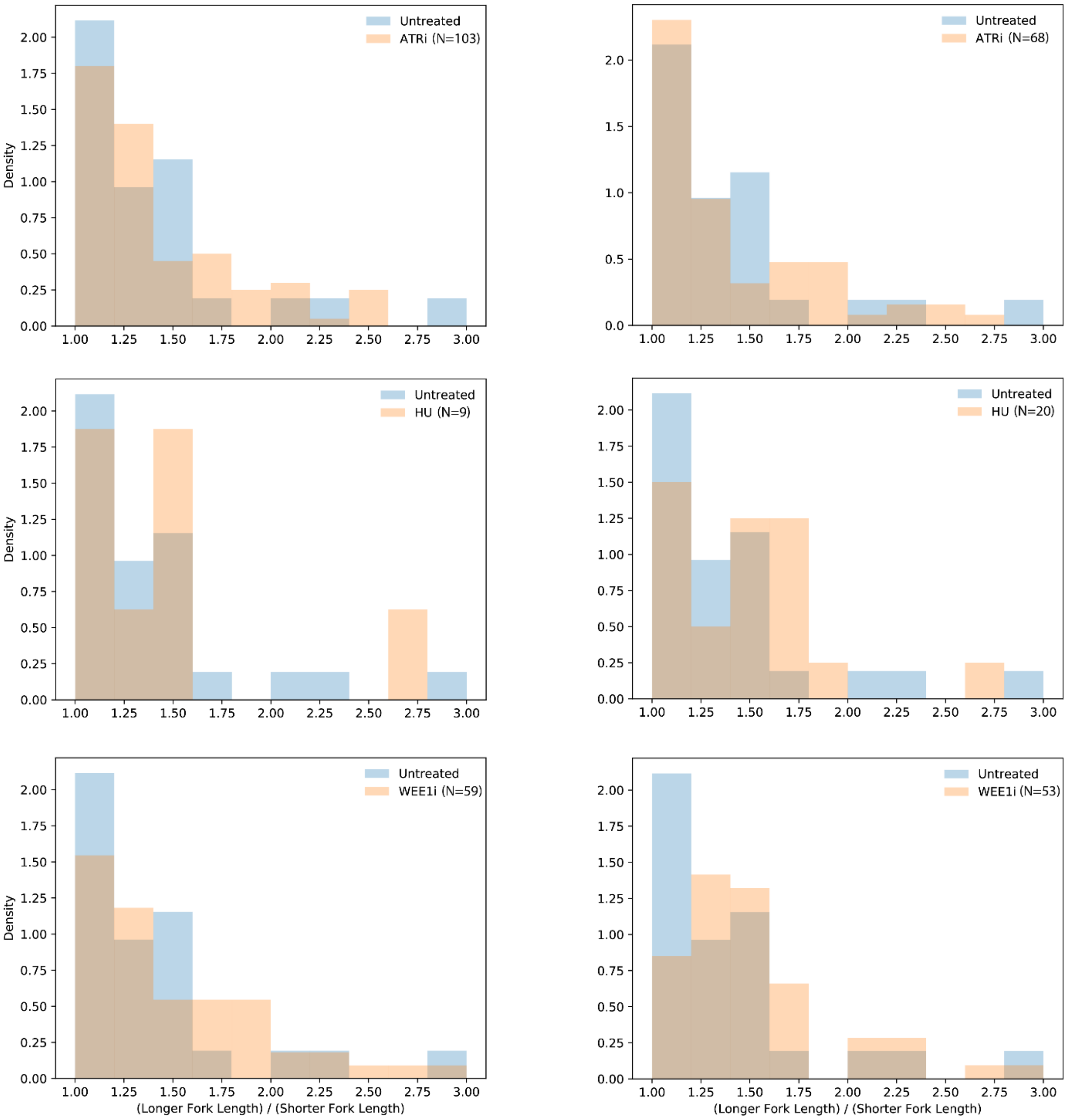

Figure S14: Asymmetry in fork track lengths at origin calls. For origin calls where the corresponding left- and right-moving fork tracks did not reach the end of the sequenced DNA molecule, the quantity (longer fork length)/(shorter fork length) was computed for each origin. While we observed an increase in asymmetry under some treatments, none of the above treated distributions (orange) showed a significant difference from the untreated distribution (blue) according to a Kolmogorov-Smirnov test. Source data are provided as a Source Data file.

Table S1

Cell Type/Treatment	Number of reads	N50 (kb)	Mean Read Length (kb)	Median Read Length (kb)	Left fork calls	Right fork calls	Origin calls	Termination calls
**A2058-Untreated**	60900	88.5	67.8	50.3	484	511	103	76
**A2058-Untreated**	133553	84.7	66.2	50.5	848	845	106	101
**A2058-ATRi**	146965	85.8	67.0	51.4	925	906	180	134
**A2058-ATRi**	216198	91.5	70.3	53.0	1275	1264	279	185
**A2058-HU**	35485	91.2	70.0	52.8	241	259	41	47
**A2058-HU**	108923	99.4	74.2	54.6	501	514	44	57
**A2058-WEE1i**	175683	98.0	73.3	54.0	1597	1672	228	213
**A2058-WEE1i**	228003	82.9	65.4	50.8	1774	1760	210	187
**A2058-PARPi**	60063	92.9	70.5	52.2	40	54	2	4
**A2058-PARPi**	262244	98.5	74.2	55.1	607	611	42	39
**HCT116-WT**	181241	89.2	68.7	51.5	2051	2135	379	363
**HCT116-WT**	168786	90.9	69.5	51.7	2454	2315	435	445
**HCT116-*CDK2*$^{AF/AF}$**	199360	86.7	66.9	49.5	2018	2016	452	350
**HCT116-*CDK2*$^{AF/AF}$**	140372	88.2	67.9	50.4	1746	1748	398	317
**RPE1-Untreated**	194322	93.4	71.7	55.3	2685	2739	456	421
**RPE1-Untreated**	209935	82.1	65.0	51.0	2767	2764	508	417
**RPE1-Untreated (R10)**	396099	74.7	60.9	50.1	5319	5320	1041	791
**RPE1-ATRi (R10)**	190016	91.9	71.6	59.4	2213	2292	495	302

Table S2

Cell Type/Treatment	Number of reads	EdU region calls	BrdU region calls	Left fork calls	Right fork calls	Origin calls	Termination calls
**RPE1-No BrdU or EdU**	4151	0	0	0	0	0	0
**RPE1-No BrdU or EdU**	106716	3	4	2	2	1	0

Table S3

Residual Block	Number of filters (f)	Kernel size (k)
Block 1	64	5
Block 2	64	5
Block 3	128	9
Block 4	128	9
Block 5	256	17

Table S1: Yields from Oxford Nanopore and DNAscent from each sequencing run. The number of reads shown for each sequencing run is the number of reads that had a mapping length to the reference genome greater than 20 kb, a mapping quality greater than or equal to 20, and passed the quality controls in DNAscent v3.1.2 (or DNAscent v4.0.3 in the last two rows). The N50, mean read length, and median read length were all calculated on this group of reads rather than on all reads from the sequencing run, as short reads will not be long enough to measure replication fork speed and stress given the 15-minute BrdU-EdU pulse used.

Table S2: False positive rates for human genomic DNA not treated with BrdU or EdU. Each row is a biological replicate, and the number of reads shown for each sequencing run is the number of reads that had a mapping length to the reference genome greater than 20 kb, a mapping quality greater than or equal to 20, and passed the quality controls in DNAscent v3.1.2.

Table S3: Neural network architecture for DNAscent v4.0.3. For each of the residual blocks in Figure S9, the number of filters and kernel size of the 1D convolution are shown. Within a block, all pointwise and depthwise convolutions use the same number of filters and kernel size.

Table S4

	DNAscent v3.1.2		DNAscent v4.0.3	
	EdU	BrdU	EdU	BrdU
P. falciparum	0.002	0.002	0.001	0.0002
H. sapiens	0.001	0.015	0.0001	0.0001

Table S5

	**no aug**	**g=4**	**g=6**	**g=8**	**g=12**
Unlabelled augmented into BrdU-labelled		20000	20000	20000	20000
Unlabelled augmented into EdU-labelled		20000	20000	20000	20000
Unlabelled	50000				
BrdU-labelled augmented into EdU-labelled		2500	2500	2500	2500
EdU-labelled augmented into BrdU-labelled		2500	2500	2500	2500

Table S4: False positive rates for DNAscent v3.1.2 and DNAscent 4.0.3. Entries in the table show the false positive rate for BrdU and EdU calls in unlabelled human and *Plasmodium falciparum* DNA. A BrdU or EdU call was considered positive if the probability from the model exceeded 0.5, and the number of positive calls was then divided by the number of thymidine positions considered by the model. In each case, benchmarks were carried out on 500 reads that passed DNAscent's default minimum read length and mapping quality thresholds.

Table S5: Number of augmented training segments used to train DNAscent v4.0.3. Number of 2-kilobase read segments used to train the model in DNAscent v4.0.3 for each data augmentation strategy. Following the training strategy of DNAscent v2 (Supplemental Figure S2 of Boemo, *BMC Genomics* 2021), 9mers were randomly augmented on average every *g* thymidines.
